# Soft 3D-Printed Phantom of the Human Kidney with Collecting System

**DOI:** 10.1007/s10439-016-1757-5

**Published:** 2016-11-09

**Authors:** Fabian Adams, Tian Qiu, Andrew Mark, Benjamin Fritz, Lena Kramer, Daniel Schlager, Ulrich Wetterauer, Arkadiusz Miernik, Peer Fischer

**Affiliations:** 1 Micro Nano and Molecular Systems Lab, Max Planck Institute for Intelligent Systems, Heisenbergstr. 3, 70569 Stuttgart, Germany; 2 Department of Urology, University Medical Center Freiburg, Hugstetterstr. 55, 79106 Freiburg, Germany; 3 Institute of Forensic Medicine, University Medical Centre Freiburg, Albertstr. 9, 79106 Freiburg, Germany; 4 Department of Radiology, University Medical Centre Freiburg, Hugstetterstr. 55, 79106 Freiburg, Germany; 5 Institute of Physical Chemistry, University of Stuttgart, Pfaffenwaldring 55, 70569 Stuttgart, Germany

**Keywords:** Kidney model, Organ phantom, 3D printing, Op-simulation, Endoscope training

## Abstract

Organ models are used for planning and simulation of operations, developing new surgical instruments, and training purposes. There is a substantial demand for *in vitro* organ phantoms, especially in urological surgery. Animal models and existing simulator systems poorly mimic the detailed morphology and the physical properties of human organs. In this paper, we report a novel fabrication process to make a human kidney phantom with realistic anatomical structures and physical properties. The detailed anatomical structure was directly acquired from high resolution CT data sets of human cadaveric kidneys. The soft phantoms were constructed using a novel technique that combines 3D wax printing and polymer molding. Anatomical details and material properties of the phantoms were validated in detail by CT scan, ultrasound, and endoscopy. CT reconstruction, ultrasound examination, and endoscopy showed that the designed phantom mimics a real kidney’s detailed anatomy and correctly corresponds to the targeted human cadaver’s upper urinary tract. Soft materials with a tensile modulus of 0.8–1.5 MPa as well as biocompatible hydrogels were used to mimic human kidney tissues. We developed a method of constructing 3D organ models from medical imaging data using a 3D wax printing and molding process. This method is cost-effective means for obtaining a reproducible and robust model suitable for surgical simulation and training purposes.

## Introduction

In order to plan, test, and practice surgical procedures before they are used in patients it is necessary to have access to organ models.^14^ Three-dimensional (3D) organ models are particularly useful in surgical urology because miniaturized medical equipment both for endoscopic (including laparoscopic) and open surgical procedures are often used. A molded soft kidney phantom using human tissue-like materials that includes an anatomically correct representation of both the collecting system and the surrounding tissues has to our knowledge not been reported previously.

Surgical simulations are already performed in operative urology and several simulator systems are already in use.^10^,^15^,^22^ However, these simulation systems typically lack anatomical details and are often fabricated from hard plastics with material properties that are very different from the target organ. Animal models, however, still play an important role in surgical training and biomedical device testing. Apart from the bioethical considerations, animal models have a number of disadvantages. These include: (1) The morphology and tissue properties of animal organs are often different from human organs; (2) The preparation is expensive and labor intensive; (3) The organs show large variations and quickly start to degrade. It is therefore important to develop realistic artificial organ phantom systems.

Recently, 3D printing technology has rapidly advanced and presents unique opportunities for the direct “printing” of organ structures.^20^ Commercial printed organ models are available.^1^ Materials of different colors are used for the surgeon to visualize important structures of the organ such as blood vessels in a liver or a kidney tumor, including individualized patient-specific 3D models.^1^ However, these printed models are often made of hard plastic materials and lack interior cavity structures found in the target organ, such as the kidney’s collecting system. Recently, 3D printing and vacuum casting were combined to manufacture an abdominal aortic aneurism (AAA) model.^5^ A silicone outer mold and a wax inner mold were created based on a 3D-printed AAA artery, and the final phantom was molded in polyurethane resin that mimics the artery’s stress–strain behavior. In 2014 Cheung *et al.* presented a pediatric pyeloplasty simulator using a laparoscopic dry-laboratory model developed with 3D-printing and silicone modeling. The intrarenal collecting system is underrepresented, so this model is not suitable for endoscopy training.^3^ Another kidney model was developed for training in fluoroscopy-guided percutaneous nephrolithotomy (PCNL) access.^19^ This model consists of a collecting system embedded into a silicone block realized by a combination of molding and 3D-printing. 3D-printed water-soluble collecting systems were dissolved from the silicone block leaving a hollow structure.^19^ The outer shape of the kidney was not considered, which makes this model not suitable for parenchymal interventions or ultrasound-based procedures. Furthermore, the fabrication is limited to non-water-soluble materials, which differ from real tissues.

Here, we present a soft phantom of the human kidney with detailed anatomical structures and consider the mechanical and physical properties of the phantom-materials. The model is based on high resolution medical CT (computed tomography) images. It is created by a technique combining 3D wax printing and polymer molding. We validated our model using CT scanning, ultrasound imaging, and endoscopy. Our fabrication method is versatile and our phantom shows mechanical and acoustic properties similar to real kidney tissue. The preparation method is inexpensive and reproducible. The phantom may be used in place of animal models. The organ model can be used to test medical devices and to simulate urological endoscopic procedures.

## Materials and Methods

### Dissection of the Kidneys

In the course of regular autopsies performed within a maximum postmortem period of 48 h on bodies stored at a temperature of 4 °C, the kidneys were removed and imaged via CT scanning. After imaging, the kidneys were returned to the autopsy room, dissected in the usual manner, and placed back into the body. Persons under the age of 18 years and those known to have suffered from a renal disease were excluded from the study. Kidneys with significant anatomical and/or pathological changes were also excluded from the study. During the procedure, the renal hilum was visualized, while the renal artery and vein were ligated and dissected distally. The ureter was also ligated and dissected in the upper third. Perirenal fat was largely excised. Three cadaveric kidneys were harvested and measured.

### CT Imaging of Human Kidneys

Iodinated contrast agent (iodine concentration of 400 mg/ml; Imeron 400; Bracco S.p.A., Milan, Italy) was injected into the collecting system via a silicone tube (12 French) connected to the ureter. Finally, the cadaveric kidney was scanned using computer tomography (Somatom Definition Flash; Siemens Healthcare) with a spatial resolution of 0.3 mm. Data were reconstructed out of the axial plane with a slice thickness of 0.6 mm, matrix size of 512 × 512, and a field of view of 154 mm × 154 mm.

### Image Segmentation, Surface Reconstruction and Design of the Mold

**Figure 1:**
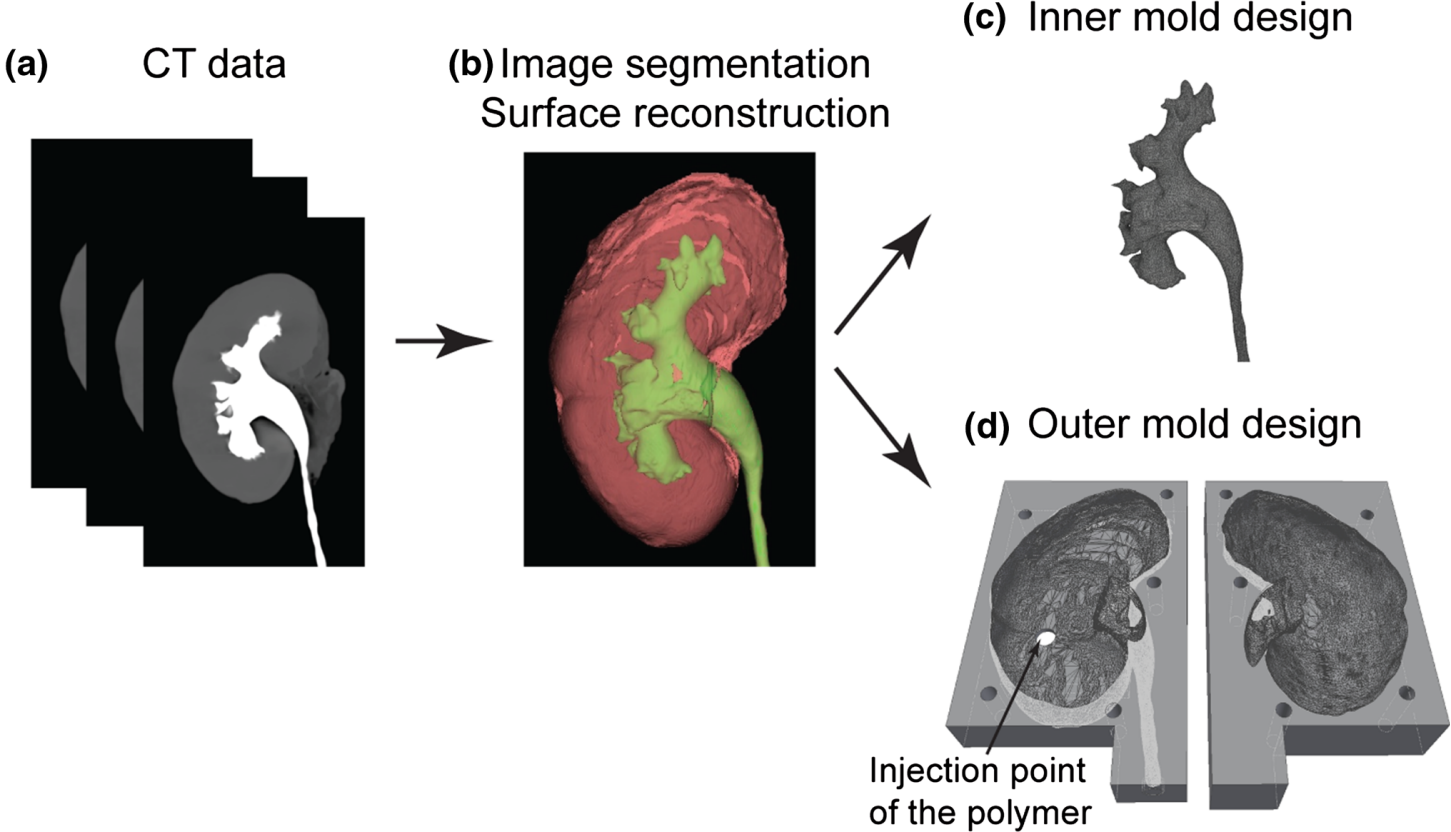
Workflow for the design of the model. (a) CT imaging data of a human kidney was acquired. (b) CT images were constructed to the 3D model. (c) The collecting system is used as the inner mold. (d) The outer shape of the kidney is used to design two separated negative molds.

The workflow developed to design the organ model from medical imaging data is shown in Fig. 1. The DICOM files were reconstructed in InVesalius 3.0.0 (Centro de Tecnologia da Informação Renato Archer, Brazil, freely-available on the web). In the CT data (Fig. 1a), the collecting system appears white due to the concentrated contrast agent, while kidney tissue is shown in gray. This contrast difference allowed the collecting system (green) and kidney tissue (red) to be reconstructed as separate 3D models (Fig. 1b). The fat tissue and unconnected debris were erased. Two freeform surfaces were represented by triangular tessellation and exported as STL (stereolithography) files, respectively.

The STL files of both the collecting system and the kidney outer shape were checked for anatomical correctness and then imported to Inventor 2016 (Autodesk), converted and stitched to a solid body using a freely-available plug-in “Autodesk Mesh Enabler”. By Boolean subtraction operation of two solid parts, two outer molds were designed and they were separated along the largest middle plane of the kidney, as shown in Fig. 1d. The ureter connects the inner mold and the outer mold and is embedded in the lower half of the outer mold. An inlet and outlet to permit the filling of the mold were added. To save 3D printing time and materials, the models used in this study were all scaled down to 80% in all three dimensions.

### 3D Printing and Molding of the Phantoms

**Figure 2:**
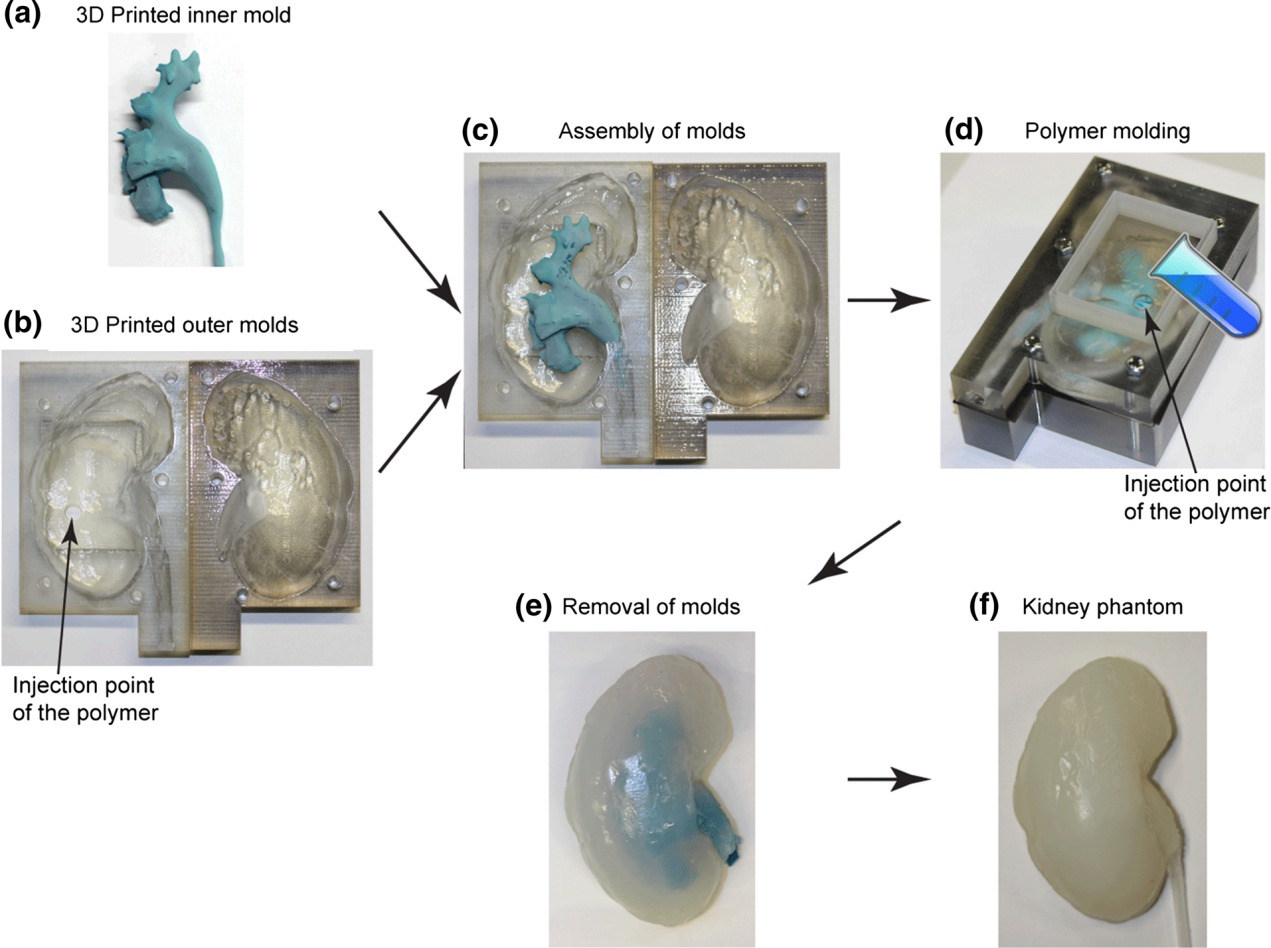
Workflow for building a 3D kidney phantom. (a) The inner mold is 3D printed in wax. (b) The outer mold is 3D printed in photopolymer. (c) The wax mold is inserted, and the upper and lower outer molds are assembled and sealed. (d) Liquid polymer is poured into the mold and degassed. (e) The phantom is demolded from the outer mold, and the inner mold is dissolved in ethanol. (f) The obtained kidney phantom.

**Figure 3:**
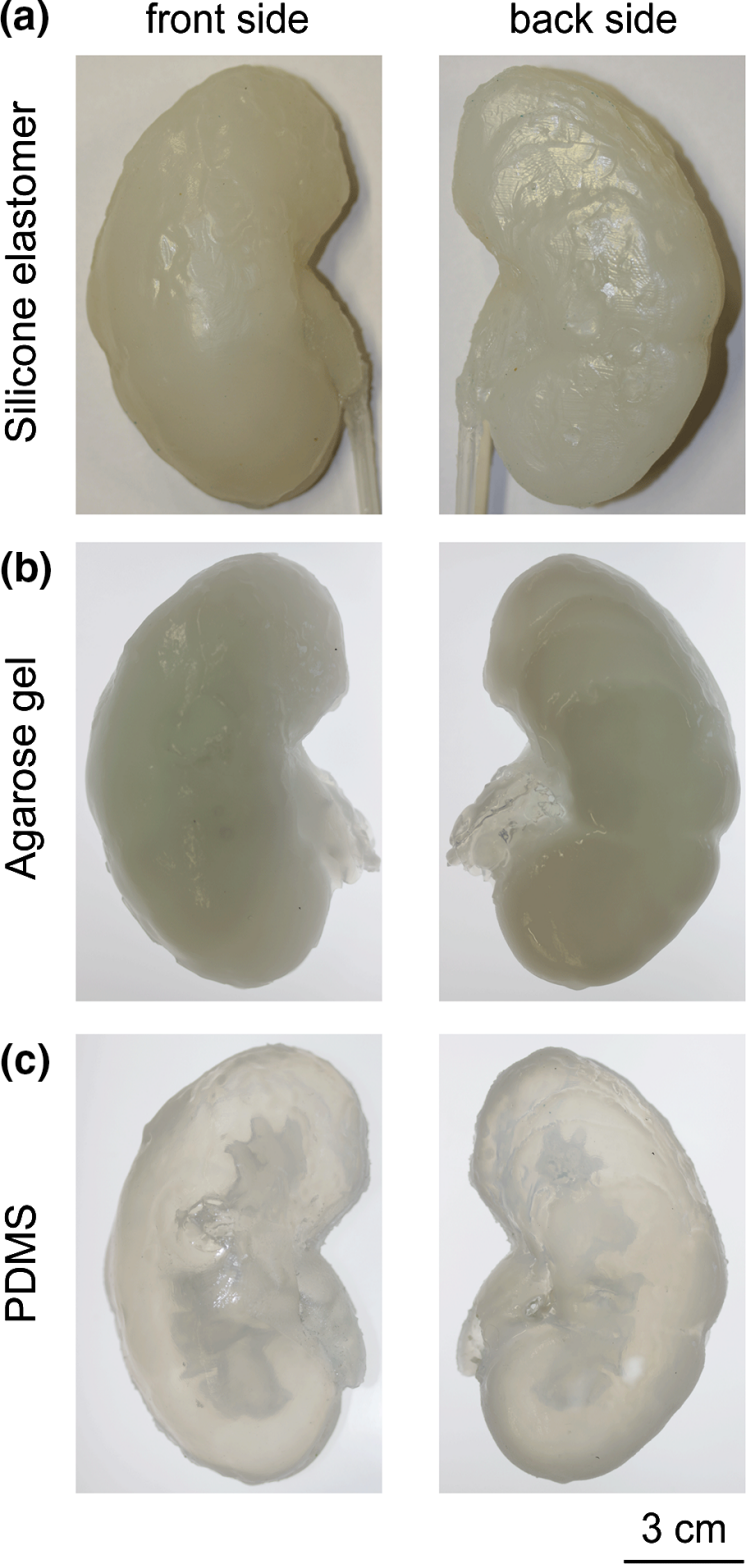
Images of the kidney phantoms made of different materials (front and back side): (a) silicone elastomer, (b) Agarose gel, (c) PDMS.

Figure 2 shows the fabrication process of the kidney phantom. The collecting system (bounding box size ~135 × 45 × 30 mm^3^) was printed out of an engineered wax material on a commercially available 3D printer (3Z pro, Solidscape, NH, USA) in ~25 h. The supporting wax was removed in 55 °C petroleum with continuous magnetic stirring on a hot plate. The outer molds were printed on a 3D printer (Objet 260 Connex, Stratasys, Israel) with a UV curable photopolymer VeroClear^®^ in high-speed mode with 32-micron layer-thickness. The two halves of the mold (bounding box size ~150 × 100 × 50 mm^3^) were printed with cavity upwards and in a glossy mode to achieve a smooth surface finish. Transparency of the material VeroClear enables the check of the filling of the mold. The printing consumed ~480 g VeroClear and ~200 g Support material, and took 4.3 h in time. The supporting material was removed using a water jet. The wax mold was then assembled into one half of the outer mold by fitting the ureter part (Fig. 2c) and fixed with a fast-curing siloxane material (No. 4667, Coltene Whaledent, Switzerland). Therefore, the inner mold was stable in the right position when the liquid polymer was poured. The outer molds were then sealed with a rubber gasket and fixed with screws. A silicone material Ecoflex (00-20, Smooth-on, PA, USA) was mixed, degassed, and poured into the assembled mold. The polymer was cured under room temperature for 2 h and then demolded from the hard mold. The inner wax material was removed by dissolving the wax in ethanol with continuous magnetic stirring at 70 °C. The phantom was then placed in an oven at 100 °C for 30 min to quickly attain maximum physical and performance properties. A silicon tube (OD = 5 mm, ID = 3 mm) was connected to the phantom to mimic the ureter and sealed with silicone adhesive. Two further models were, respectively, made of agarose gel (Agarose Electran, VWR), and polydimethylsiloxane (PDMS) (Sylgard 184, Dow Corning) (Fig. 3). After dissolving the wax in ethanol, the phantom was thoroughly rinsed with water. The phantom made of agarose gel was stored in water in a fridge, which ensured the usability of the phantom for at least 6 months. Ten kidney phantoms were fabricated in total.

### CT Imaging and Validation of the Phantoms

**Figure 4:**
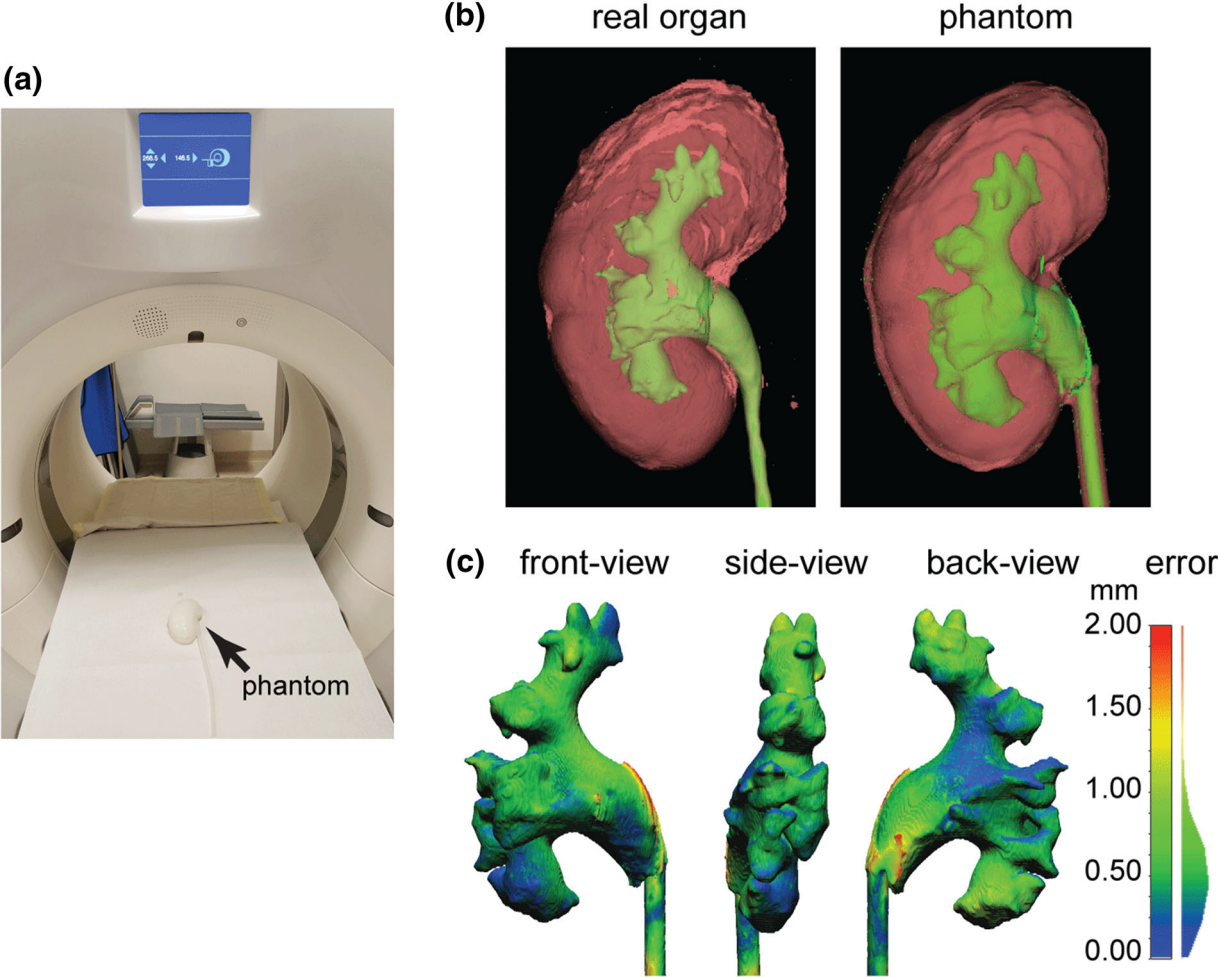
Evaluation of the accuracy of the phantom. (a) The phantom (silicone elastomer) in a CT scanner. (b) Qualitative comparison of the two 3D-reconstructions. The phantom in Ecoflex material replicates the structure of the real organ with detailed features. Kidney tissue is shown in red, and the collecting system is shown in green. (c) Quantitative error analysis of the collecting system in the phantom when compared to the original CT scan. The surface color of the phantom model represents the distance error, compared to the real organ as a reference. Note the distribution of the error is shown on the right side of the color bar, with the maximum error of 2 mm and a mean error of around 0.6 mm.

The silicone elastomer phantom was imaged without contrast agent using the aforementioned CT scanner. From the obtained CT images (Fig. 4a), the collecting system and the outer shape of the phantom were reconstructed, up-scaled to 125% to the original scale of the organ, and the mesh density was high enough thus the error introduced during the digital scaling process can be neglected. A detailed quantitative analysis was made of the inner structure by determining the differences between the phantom and the CT model using a 3D triangular mesh editing software (CloudCompare v2.6.1). Two separate meshes in the STL files were manually aligned by selecting three marker points in each mesh. The three marker points are selected to be the relatively sharp endpoints on the surface of the collecting system, which can be easily recognized as alignment markers. After the definition of each of the three marker points the software overlays the meshes of the real organ and of the phantom, such that a Cloud/Cloud distance can be computed. Three phantoms made of three different materials were independently validated by this process. Figure 4c shows a difference map between the tissue-like elastomer phantom made from Ecoflex material and the real kidney and it is seen that the error is negligible. CT scans were also performed on the phantoms made of the other two materials used in this study and similar mean errors of ~ 0.6 mm were measured.

### Ultrasound Examination of the Phantom

**Figure 5:**
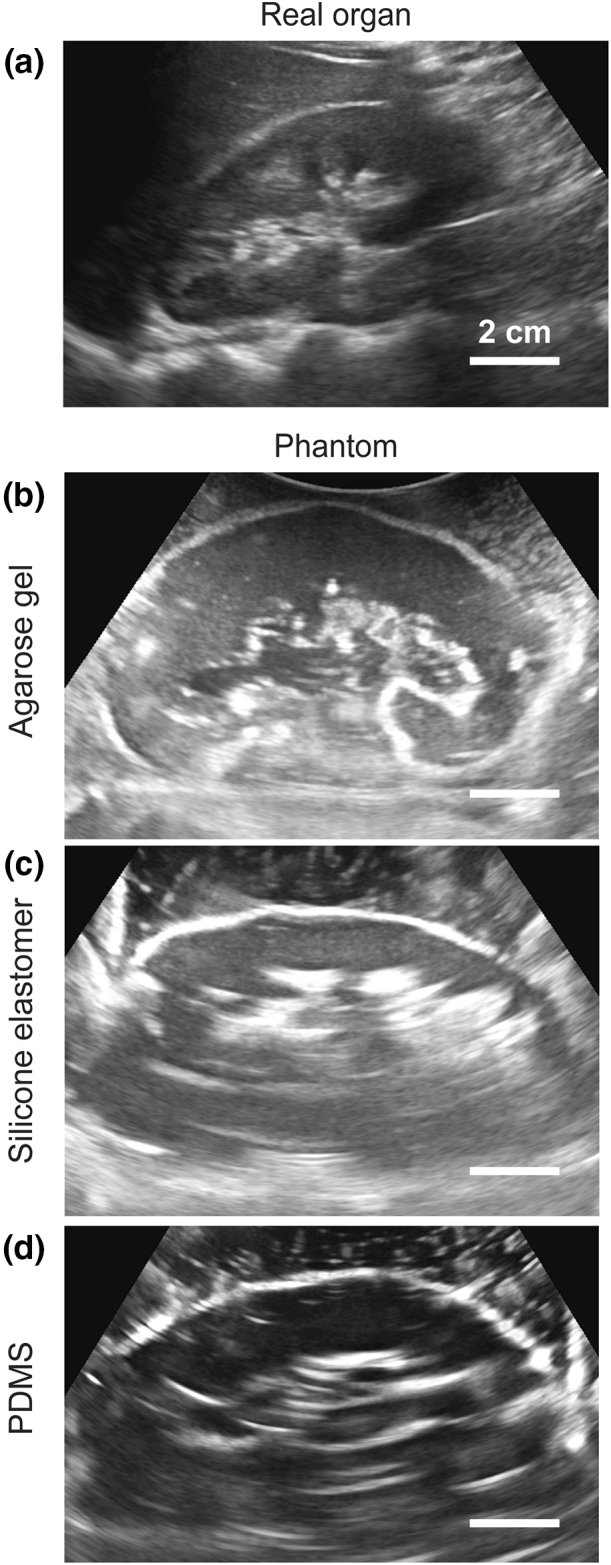
Ultrasound images of the three models made of different materials in comparison to a real human kidney. The agarose model outperforms the other types of materials in terms of replicating the outer shape and tissue of the kidney, especially the appearance of the collection system, when compared to the real organ.

The three kidney phantoms were examined with a commercial ultrasound system (HI Vision Avius^®^; Hitachi Ltd.) using standard abdomen settings (B-mode). First, the kidney phantoms were fixed in the center of a 15 cm × 30 cm water filled container to mimic the ultrasound propagation through the abdomen. The model was presented in the coronal plane and the convex ultrasound transducer was placed approximately 2 cm below the water surface. The distance between the ultrasound transducer and the phantom was approximately 4 cm. Figure 5 shows ultrasound images of the three different phantom materials and that of a real kidney from a living human.

### Endoscopic Validation of the Phantom

A flexible 10 French ureterorenoscope (Richard Wolf, Knittlingen, Germany) was used to endoscopically examine the collecting system of the models. The endoscope was connected to a 5520 1CCD Endocam and a 5132 Auto LP Highlight Module (Richard Wolf, Knittlingen, Germany) visualized by a LCD monitor (Sony, Tokyo, Japan). The endoscopic video was recorded by a computer and a snapshot is shown in Fig. 5. During the endoscopic procedure a constant water flow (0.5 ml/s) was maintained.

## Results

### Anatomical Structure Comparison of the Phantoms and the Original Kidney

#### CT Validation of the Phantoms

As shown in Fig. 4, the outer shape of the kidney phantom closely matches that of the 3D reconstructed model (Fig. 4b). To confirm that the inner structure of the phantom indeed replicates the real kidney, a second CT scan was performed on the phantom with the same parameters as those used on the real kidney (Fig. 4a). Both the renal pelvis and all calyces corresponded to the respective structures of the CT image of the cadaveric kidney by radiological criteria. The CT reconstruction showed that the molding process successfully reproduced morphological details of the collecting system to below 1 mm, which was only limited by the resolution of the original CT scan. From the reconstruction of the 3D model, it is clear that the inner and outer surfaces of the model closely represent those of the original organ (Fig. 4b). Comparing with the real kidney, the collecting system of the phantom is in a slightly lower position relative to the surrounding kidney tissue with an offset of ~ 4 mm in Z direction, which is due to the error introduced during the assembly of the inner mold into the outer mold. A better fitting geometry that limits the relative positions of the two molds at the ureter part can be designed in future to improvement the absolute fit.

Quantitative comparison between the collecting systems of the phantom compared to the real organ showed a maximum error of 2 mm. Computations of both “point to point” and “point to triangle” distances were calculated using different local model methods. The results shown that the mean distance error is independent of the calculating method, and equals 0.6 mm for the total collecting system (with a bounding box dimension of approximately 135 mm [length] × 45 mm [width] × 30 mm [height]). Thus, the mean error of the model is about 1%, which is suitable for endoscopic training and testing purpose. The largest error (represented in red in Fig. 4c) was concentrated mainly in the pelvis, but the more detailed structures of the calyces are all well replicated. This suggests that the major error was most likely due to misalignment of the inner mold with the outer hard mold and not from the image reconstruction or the 3D printing process. Ten kidney phantoms were manufactured in total using three different materials, and one phantom for each kind of material was scanned by CT, reconstructed and the error in distance was calculated. The statistics show that most distance error fall in the range 0.3–0.7 mm, and the mean error for three phantoms is 0.6 mm.

#### Ultrasound Validation of the Phantoms

Compared to the real kidney (Fig. 5a), the agarose gel model outperforms the three other materials as it closely resembles the acoustic impedance of human tissues.^23^ This phantom represents the outer shape of the kidney, the tissue, and especially the shape of the collection system with particularly good detail (Fig. 5b). Due to the differences in the elasticity of the materials, the models made of silicone elastomer and PDMS displayed a strong signal at the outer surface, however, only a white outline of the phantom can be seen in Figs. 5c and 5d.

#### Endoscopic Validation of the Phantoms

**Figure 6:**
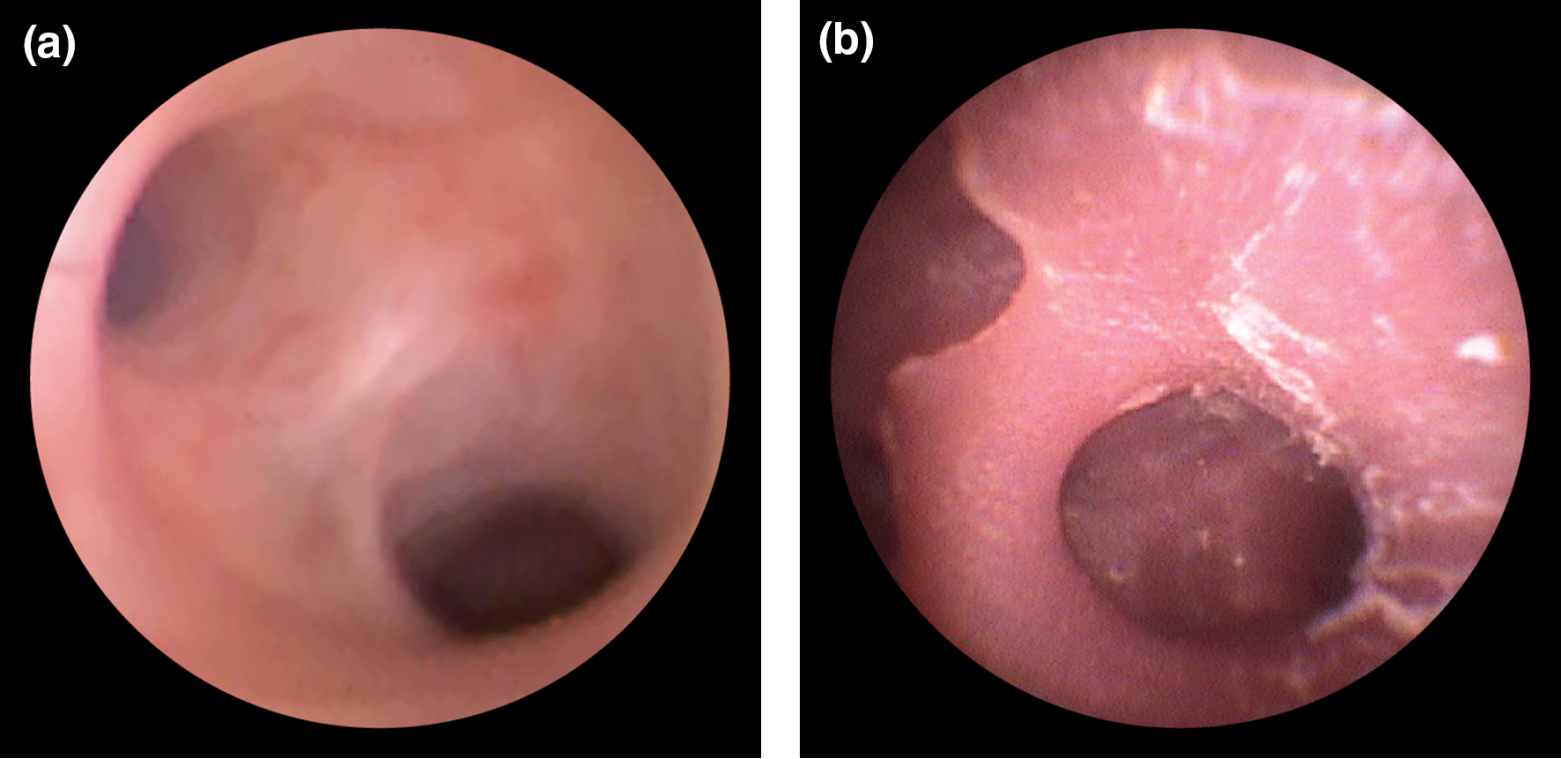
Endoscopic validation. The view of upper calyces in a real human kidney (a) and in the silicone elastomer phantom (b).

We also performed an endoscopic assessment by conventional flexible ureterorenoscopy. On the inside of each phantom, a smooth surface that represents the typical morphological characteristics of the upper urinary tract was visualized endoscopically (Fig. 6). Aside from the whitish color of the polymer differing from the color of real tissue, the complete collecting system appeared visually identical to a human kidney. All calyces were easily intubated with the 10-French flexible ureterorenoscope. The spatial orientation of the instrument was clear at all times. Of the three materials evaluated, the transparent PDMS model offers new possibilities for endoscopic training and device testing purposes.

#### Comparison of the Material Properties of the Phantom and Kidney Tissue

The advantage of this work, when compared to current urological teaching and training systems and other previously reported 3D printed kidney models,^1^ is that our method permits a wider variety of materials to be used, including those that have a similar elastic modulus to kidney tissue—the elastic modulus of porcine kidney is 48.56 ± 7.32 kPa.^13^ Table 1 summarizes the material properties of the three molding polymers used to build our phantoms compared to the direct 3D-printable materials TangoPlus^®^/TangoBlackPlus^®^ (Stratasys, Eden Prairie, MN, USA).

**Table 1:** We compared the material properties of the three polymers used to replicate kidney tissue in this study, as well as, TangoPlus^®^/TangoBlackPlus^®^ (directly 3D printable elastomers).

Materials	Kidney tissue	Silicone elastomer	Agarose (4%)	PDMS	TangoPlus^®^/TangoBlackPlus^®^
Shore hardness	–	20 (type 00)	60–70 (type 00)^12^	44–54 (type A)^7^	26–28 (type A)^18^
Elastic modulus (kPa)	49^13^	60	49^2^	1320–2970^7^	965–1051
Tensile strength (MPa)	4–9^16^	1.1	0.3–0.5^11^	3.51–7.65^7^	0.8–1.5^18^

We found that the elastic modulus of TangoPlus^®^/TangoBlackPlus^®^ was approximately 20 times higher than real kidney tissue. In addition, the material was completely opaque. The silicone elastomer had an elastic modulus of 60 kPa, which was very close to that of real kidney tissue. Furthermore, the material had a relatively low viscosity of 3 Pa s in the uncured liquid state, which permits fine structures to be molded. PDMS (Sylgard 184, Dow Corning) is a popular polymer that has been used in microfluidics^21^ and in building artificial organ systems.^8^,^17^ It shows excellent optical transparency. This facilitates a clear visualization of the collecting system inside the kidney from the outside, which could be valuable for endoscopic training. However, the elastic modulus of PDMS is much larger than that of real kidney tissues. Agarose is a polysaccharide polymer material that is easy-to-prepare and biocompatible, thus it has been widely used as a material to mimic soft tissues for magnetic resonance imaging (MRI)^6^ and ultrasound imaging.^4^ As the concentration of the agarose gel can be varied, the elastic modulus can be tuned to match the elastic modulus of real kidney tissue. Although these three materials may not fully represent all mechanical properties of the biological tissues (e.g. they exhibit lower tensile strengths), the materials serve as proof-of-concept examples to show the scheme reported in this paper is general and can be applied to a variety of polymer materials and their mixtures. In the future, more materials can be engineered in order to mimic specific details and properties of biological tissues.

## Discussion

The fabricated kidney phantoms accurately represent the kidney’s shape and important anatomical structures, such as the collecting system. Our presented method of 3D printing and molding exhibits two major advantages: (1) It allows the use of a wide variety of materials, e.g. transparent materials for research and training, very soft materials mimicking human tissues, including water-soluble and non-toxic biocompatible materials for tissue engineering. In previous works, polyvinylalkohol (PVA) was used for the cavity structures and dissolved in water which calls for a non-water-soluble phantom material.^19^ Acrylonitrile–butadiene–styrene (ABS) material was also used, but had to be removed with the toxic solvent xylene.^9^ Differed from these works, we use hot ethanol to fully dissolve the wax material, which allows the usage of water-based gels and does not cause any shrinkage or damage to the gels. Water based gels also exhibit more realistic tissue-like properties, which is important for the simulation of medical procedures, such as the ultrasound imaging shown in Fig. 5 in this paper. Agarose gel outperforms other materials. As the water content is high and the concentration can be varied, the density, elasticity, electrical and acoustic impedance can all be tuned to resemble the corresponding properties of human tissues.^23^ Moreover, micro/nanoparticles can be readily mixed in the gel, thus the heterogeneity of the tissues may also be simulated. (2) Complicated 3D shapes, especially inner cavities can be precisely built with our method. The wax material can efficiently be removed from the mold even though the calyces are connected by small openings to the kidney pelvis. Differences between the real kidney and the model are not due to the 3D printing or molding process, but arise when the collecting system is placed into the outer mold. A more detailed design of the connection part can reduce this small error. Nevertheless, the anatomically important regions show a mean error of only 0.6 mm.

One drawback of the current workflow is that it takes 20 h to print the wax mold, 4 h to print the hard mold, 2 h for the polymer to cure and another 5 h for demolding and dissolving the wax. In total, the whole process takes about 2 working days to start from a 3D model to a ready-to-use phantom. However, most of the procedures are automated, the work is not labor intensive and the time is mostly spent waiting.

The phantoms reported herein exhibit several advantages including longer durability, robustness, and reproducibility when compared to animal organ models. Our phantom also outperforms existing organ phantoms as it more accurately replicates the anatomy of a human kidney and its mechanical properties. The phantoms can be used in future experiments to simulate percutaneous diagnosis and treatment methods like a biopsy or percutaneous nephrostomy, respectively. It is also conceivable that this model would be helpful in the testing and development of imaging techniques, such as ultrasound, magnetic resonance imaging (MRI), or computed tomography.

## Conclusions

A soft human kidney phantom with detailed anatomy, including the pyelocaliceal system, is built by a novel fabrication process-combining 3D printing and polymer molding. The method is versatile since it replicates anatomical details with sub-millimeter resolution and permits a wide range of materials to be used, including biocompatible hydrogels. We foresee a number of applications for the kidney phantom, including surgical planning, simulation and training of urological endoscopic procedures, and medical device testing.

## Abbreviations

ABS: Acrylonitrile-butadiene-styrene
B-mode: Brightness modulation
C: Celsius
CT: Computed tomography
Cm: Centimeter
3D: Three dimensional
DICOM: Digital Imaging and Communications in Medicine
ERC: European research council
FA: Faculty assistant
Fig.: Figure
h: Hour(s)
kPa: Kilopascal
Ltd.: Limited company
LCD: Liquid crystal display
mg: Milligram
min: Minutes
ml: Milliliter
mm: Millimeter
MN: Minnesota
MPa: Megapascal
MRI: Magnetic resonance imaging
NH: New Hempshire
Nr.: Number
PA: Pennsylvania
Pa: Pascal
PCNL: Percutaneous nephrolithotomy
PDMS: Polydimethylsiloxane
PVA: Polyvinylalkohol
s: Second
S.p.A.: Società per Azioni
STL: Stereolithography
USA: United States of America
UV: Ultraviolet
